# Ultrafast Nonadiabatic Electron Relaxation Dynamics in Photoexcited C_60_ Molecules

**DOI:** 10.1021/acs.jpca.4c06109

**Published:** 2025-02-20

**Authors:** Esam Ali, Mohamed El-Amine Madjet, Ruma De, Matthew B. Wholey, Thomas Frauenheim, Himadri S. Chakraborty

**Affiliations:** † Department of Natural Sciences, Dean L. Hubbard Center for Innovation, Northwest Missouri State University, Maryville, Missouri 64468, United States; ‡ Department of Physics, Faculty of Science, University of Benghazi, Benghazi 9480, Libya; ¶ Bremen Center for Computational Materials Science, University of Bremen, 28359 Bremen, Germany; § School of Science, Constructor University, Campus Ring 1, 28759 Bremen, Germany; ∥ Institute for Advanced Study, Chengdu University, Chengdu 610106, China

## Abstract

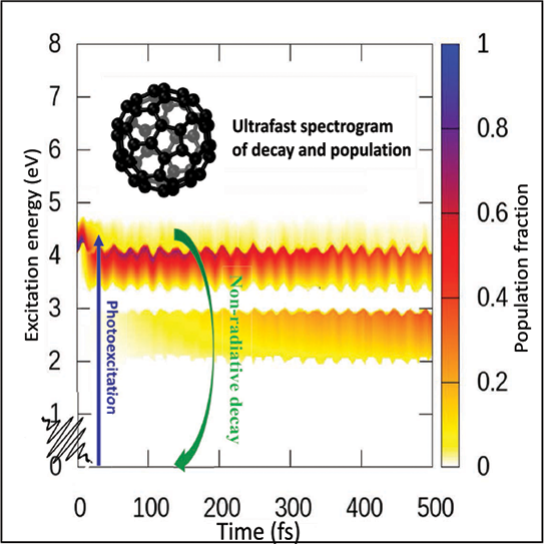

Fullerene molecules, being attractive for fundamental research and key building blocks in materials of energy harvesting, are important for ultrafast electron transfer studies. The nonradiative electron-relaxation dynamics in a C_60_ molecule is investigated after chosen initial photoexcitations. The methodology includes nonadiabatic molecular simulation combined with time-dependent density functional theory and a semiclassical surface hopping approach. Results treating the exchange-correlation by using hybrid functionals, Becke three-parameter Lee–Yang–Parr (B3LYP) and Perdew–Burke–Ernzerhof (PBE0), are presented. Both approaches produce similar unoccupied band structures in the ground state that qualitatively agree with our many-electron excited state calculation. The model-dependent differences in the ultrafast population dynamics, including the transient entrapment of the population, are studied systematically. The trend of the results demonstrates a universal dependence on the structure of the unoccupied band offering a spectroscopic route to probe the structure. Predictions can be assessed by comparison with ultrafast transient absorption or time-resolved photoelectron spectroscopy measurements. By selectively comparing with inexpensive nonempirical PBE results, the study facilitates method optimization for future studies of technologically important and larger fullerene complexes.

## 1 Introduction

Trends in technology utilize fullerene-based materials, due to their stability and unique properties, through applications in energy storage^1^ and conversion.^2,3^ For instance, pump–probe ultrafast transient absorption spectroscopy (UTAS) measurements of blends of a low-band gap polymer with fullerene derivatives found an efficient charge generation process compared to that in the pristine polymer.^4^ UTAS has also been applied to assess the effects of cage-symmetry on the electron dynamics in metallofullerenes, which are crucial for visible-light solar energy harvesting.^5^ Likewise, to aid ultraviolet (UV) harvesting for solar cell design, the UTAS approach was undertaken to study charge transport and electron transfer in a PbI_2_/C_60_ heterojunction, which demonstrated superior charge extraction efficiency.^6^ On the other hand, two-photon time-resolved photoelectron spectroscopy (TRPES) has been applied to disclose the optical generation of noninteracting excitons in a fullerene film, leading to the redistribution of transport levels in the nonexcited molecules.^7^ Therefore, ultrafast dynamics of charge separation, migration, transport, transient trapping, and recombination of photoexcited electrons in these materials are important processes.

Charge transfer (CT) is the key subprocess that underpins the core mechanism of organic photovoltaics. The donor–acceptor complexes in this application are abundantly composed of fullerene materials. A fullerene molecule can be structurally upgraded to attend to desired chemical properties in general. This can be achieved by, namely, choosing its endohedral species^8^ and/or exohedral ligands or polymers.^9,10^ The objective is to control the light absorption efficiency and carrier transport. An excitonic state is created upon absorbing a photon from the complex. This exciton either dissociates into free carriers or recombines to decay depending on the dominance of, respectively, the electron–hole separation energy or excitonic binding energy. The dissociation is the preferred mechanism for photovoltaics,^7^ the probability of which may enhance as the decay times of excited electrons elongate. Therefore, the decay and transfer of a hot electron from one location of the molecular material to another is a fundamental subprocess of these events.^6,11−14^ In contrast, a relatively quicker relaxation may favor the repopulation of cold electrons and subsequent thermalization of the molecular lattice. The latter process has applications in photothermal cancer therapy. In fact, a high photothermal efficiency and superior stability are found in polyhydroxy fullerenes for it to become an ideal candidate for such applications.^15^

Since fullerene molecules constitute the key moiety in these compounds, the understanding of these compounds’ CT processes will benefit by investigating electron relaxation in such a molecule. Besides, there is another fundamental importance. Experimentally, UTAS^16−18^ and TRPES,^7,19^ using femtosecond (fs) pulses or, more recently, attosecond pulses for greater resolution,^20,21^ can probe such dynamics in real-time. Indeed, photoinduced charge migration has been measured in the time domain for fullerene-based polymerized films^14^ and heterojunctions,^6^ and also for bulks^22^ and nanorods.^23^ Pristine C_60_ is relatively easily available to conduct such measurements. These ultrafast processes occur on the fs time scale and are driven by the strong coupling between ionic and electronic degrees of freedom. Therefore, computationally, frameworks based on nonadiabatic molecular dynamics (NAMD) are appropriate for accurate, comprehensive descriptions of the processes.^11,24^

In this study, the NAMD results are obtained employing hybrid Becke three-parameter Lee–Yang–Parr (B3LYP) and hybrid Perdew–Burke–Ernzerhof (PBE0) exchange-correlation (xc) functionals within the scope of density functional theory (DFT). In general, hybrid functionals are well-known to produce accurate results.^25,26^ In particular, B3LYP has earlier been widely engaged with success in applications of fullerene materials^4,14,27,28^ and thus is expected to be reliable in the current study. In fact, our previous publication^29^ has established a successful methodology on the B3LYP track. On the other hand, instances exist where PBE0 was found to improve upon B3LYP.^30^ Our B3LYP and PBE0 models yield unoccupied level structures closely similar, while both qualitatively match the many-electron excited spectrum that we also calculate. Comparisons of the simulations with future measurements will examine the relative accuracy of the xc models. We further compare some results from a significantly inexpensive (nonhybrid) PBE functional to capture robust effects. The resulting knowledge will facilitate the optimization of our computational methods to address complex, larger systems.

## 2 Description of Methods

The software packages and the computation workflow follow our previous study of the Mg@C_60_^29^ molecule and references therein. Gamess-US^31,32^ was used for the ground-state geometry optimization of C_60_ conducted at the B3LYP/6-311+G** level of theory. This produced a good description of the band gap, 2.7 eV, close to reference values.^33,34^ Moreover, the calculated difference of 5.15 eV between the C_60_ ionization energy and electron affinity closely agreed with the difference of these quantities measured (4.9 eV), respectively, by electron impact mass spectrometry^35^ and high-resolution photoelectron imaging.^36^

The molecular dynamics (MD) simulations were conducted on the optimized structure of C_60_ for 6000 steps with a step size of 0.5 fs. The simulations were performed in the NVT canonical ensemble at 300 K using a velocity-rescaling thermostat to maintain the temperature. Subsequently, a production run was performed in the NVE canonical ensemble, extending for another 3000 fs with 0.5 fs a step. All MD simulations were carried out using the CP2K software.^37^ As mentioned earlier, three different choices of exchange-correlation functional were employed: (i) nonempirical (nonhybrid) PBE, (ii) (hybrid) PBE0, and (iii) (hybrid) B3LYP. These functionals were used throughout the simulations until the corresponding final results were obtained. PBE is one of the preferred generalized gradient approximation (GGA) functionals, while PBE0 retains the PBE correlation part but hybridizes the exchange between Hartree–Fock (HF) and PBE in 25–75%. On the other hand, B3LYP mixes 81% LYP with 19% local spin density approximation (LSDA) for the correlation, while for the exchange, it mixes 20% HF with 80% LSDA and 72% Becke. The time-dependent populations were obtained by averaging over 20 initial configurations, and 1000 stochastic realizations of surface hopping trajectories were performed for each configuration. The DFT-D3 dispersion correction of Grimme is used to account for the dispersion interactions.^38,39^ The QMflows-namd module^40^ interfaced with CP2K is employed to compute electronic structure properties of molecular orbitals, energies, and electron–phonon nonadiabatic couplings (NACs) between orbitals to construct the vibrionic Hamiltonian; see Subsection 3.3 for more details. The early thermal equilibration phase of NVT is important to obtain accurate NACs. The energies and NACs are then used to perform the NAMD simulations using the PYXAID package; for details, see refs (41−44). Note that the energies, band gaps, and various orbital isosurface images plotted are from the last step of the NVT simulation, unless stated otherwise, to approximately represent the equilibrium ensemble.

The methodology relies on an effective independent particle (IP) framework. Although DFT does not include the nonlocal interaction *exactly* as in the pure Hartree–Fock approach, it may include an exchange-correlation interaction at various levels of mixing; see above. This results in virtual single-electron molecular orbital states. In the IP approximation applied to our study, the electronic excited state properties, such as the energies and nonadiabatic couplings, are calculated using the properties of these single-electron orbitals such as the Kohn–Sham orbitals.^41^ However, in order to examine whether the important IP characteristic of the unoccupied spectra survives in a many-body (MB) frame, we employed a description of many-electron excited states as follows. CP2K was again used to perform the MD simulations and the electronic structure calculations and to compute MB excited state energies. Consistent with previous configurations, the xc functional was augmented with Grimme’s DFT-D3 dispersion correction. In the DFT calculations, a double-ζ shorter-range basis set (DZVP-MOLOPT-SR-GTH)^45^ to represent the molecular orbitals and a plane wave basis with a 600 Ry cutoff for the electron density were considered. The Goedecker-Teter-Hutter (GTH) pseudopotentials^46^ were used to describe the core electrons for the carbon atom. We enclosed the fullerene molecule in a 20 Å wide cubic box under open boundary conditions. Additionally, the auxiliary density matrix method with the cpFIT3^47^ fitting basis set was employed. All calculations are performed with a single *k*-point, the Γ point. The state energies are computed in both IP (KS-DFT) and MB (TDDFT) levels, with the first 150 MB excited states calculated using the B3LYP xc functional.

The neglect of back-reaction approximation (NBRA) is used, in which the evolution of the nuclei is not affected by the electronic state transitions. For C_60_, this approximation is justified by a relatively rigid structure of the molecule and by the absence of significant structural changes upon photoexcitation (at least for energies considered in the present study). In fact, it has been shown in the MB frame^48^ that if the ground and first excited state potential energy surfaces are nearly parallel to each other along the propagated trajectory, then this indicates a validation of the NBRA approximation.

## 3 Results and Discussion

### 3.1 LUMO+*n* Structures

**Figure 1:**
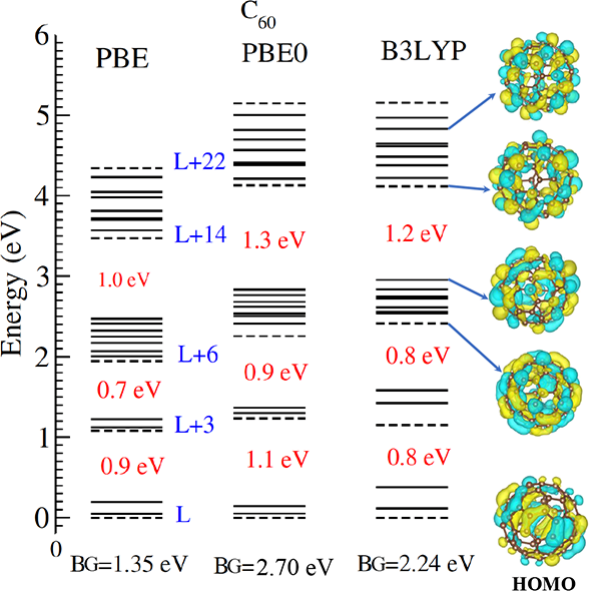
C_60_ unoccupied molecular orbital energies, relative to LUMO (L) up to LUMO+22 calculated at the DFT level of theory with the PBE, PBE0, and B3LYP exchange-correlation functional. The HOMO–LUMO band gap (BG) energy determined by each method is noted. Other energy gaps (values quoted) inlay the structures, which are qualitatively identical for the methods while quantitatively somewhat different. DFT/B3LYP generated isosurface plots of LUMO+20, LUMO+14, LUMO+13, LUMO+6, and HOMO orbitals are illustrated. All information in this figure is taken from the last step of the NVT simulation.

Figure 1 displays the unoccupied molecular orbital energies, referenced from the lowest unoccupied molecular orbital (LUMO) level, of C_60_ up to LUMO+22 calculated using the PBE, PBE0, and B3LYP functionals. PBE0 and B3LYP functionals are seen to yield closely identical energy structures. Nonhybrid PBE energies, on the other hand, are quantitatively somewhat compressed overall, but the structure is qualitatively similar. The highest occupied molecular orbital (HOMO) to LUMO band gap is smaller in PBE compared to its values in PBE0 and B3LYP which are roughly similar. Also notice the three energy gaps that open up below LUMO+3, LUMO+6, and LUMO+14 in all three sets, indicating a broad universality of the results irrespective of the choice of an xc functional. We note that such intermittent gaps in C_60_ unoccupied levels were found in other calculations as well.^29,49^ We also include the HOMO and a few selected LUMO+*n* isosurface orbital images in this figure. This displays a visual and qualitative idea of how the electron character is expected to alter from HOMO and evolve through relaxation.

**Figure 2:**
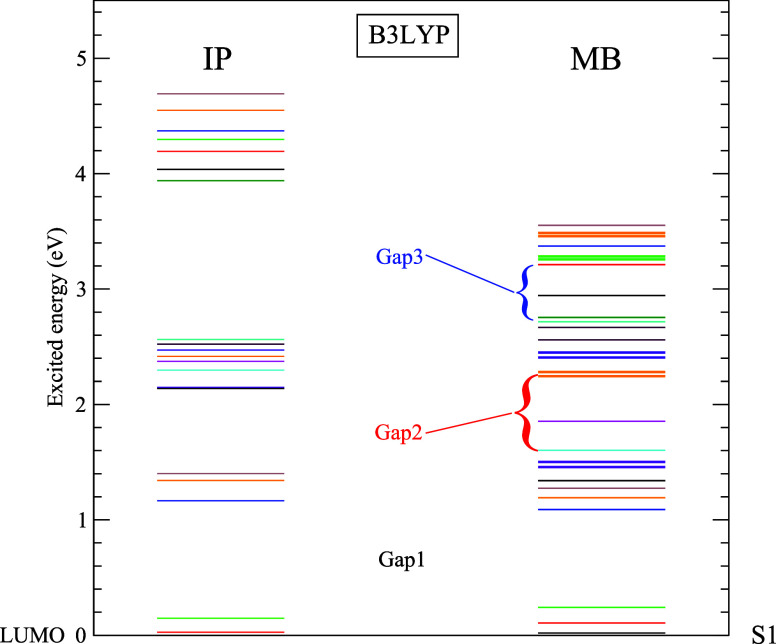
Excited state spectrum of C_60_ in the many body (MB) calculation, S1 being the first excited state, is compared to its independent particle (IP) counterpart, both based on the B3LYP functional. The IP energy gaps (BG, Gap1, Gap2, and Gap3) are well reproduced in the MB result.

In Figure 2, the calculated excited state spectrum in MB is compared to its IP counterpart obtained within the same framework. Note that addressing the hole relaxation dynamics is not our primary intention here. Hence, in the IP part, we consider the active space where electrons are excited from HOMO to LUMO+*n* states and then focus mainly on the electron relaxation from LUMO+*n* to LUMO, neglecting the electron–hole recombination. Consequently, we choose the LUMO energy as the reference in IP results and the first MB excited state (S1) energy to be the reference in MB results in Figure 2. Notice that the IP spectrum in this figure is similar to the B3LYP spectrum presented in Figure 1. Remarkably, the MB spectrum reproduces the lowest energy gap (Gap1) very well and the next higher gap (Gap2) reasonably well, although Gap3 is only qualitatively reproduced. Of course, a large number of satellite excitations being nearly degenerate in MB makes the spectrum appear denser, including the occurrence of some isolated states inside Gap2 and Gap3. However, the overall similarity provides confidence in the dependability of the following relaxation results within the IP frame, given that the simulation of full MB dynamics is prohibitively expensive. We may note however that the simulation of nonradiative relaxations in some nanocrystals suggested accelerated dynamics in the MB frame versus IP.^48,50^ Be that as it may, our previous study^29^ employing a configuration-interaction description of MB effects in Mg@C_60_ indicated that the MB dynamics, which dominates the plasmon-driven ionization spectra^51^ at extreme UV energies around 20 eV, plays a minimal role in the near to far UV energy region of current interest. Reference (29) quantitatively demonstrated that the dynamics in these regions are predominantly driven by electron–phonon couplings, reasonably modeled within the IP framework.

In any case, these energy gaps among the excited states will produce strong transient events in the dynamics that we reveal below. Electrons from the HOMO level may be photoexcited by UV pump pulses across the HOMO–LUMO band gap to selected LUMO+*n* excited states. These excited states are treated as the initial states in our simulation. Nonradiative population decay, driven by electron–phonon couplings, of these states then becomes the dominant decay process. Note that no Auger-type decay channel exists in this process since the excitation energy is lower than the first ionization threshold of C_60_.

### 3.2 Ultrafast Evolution of Populations Dynamics

**Figure 3:**
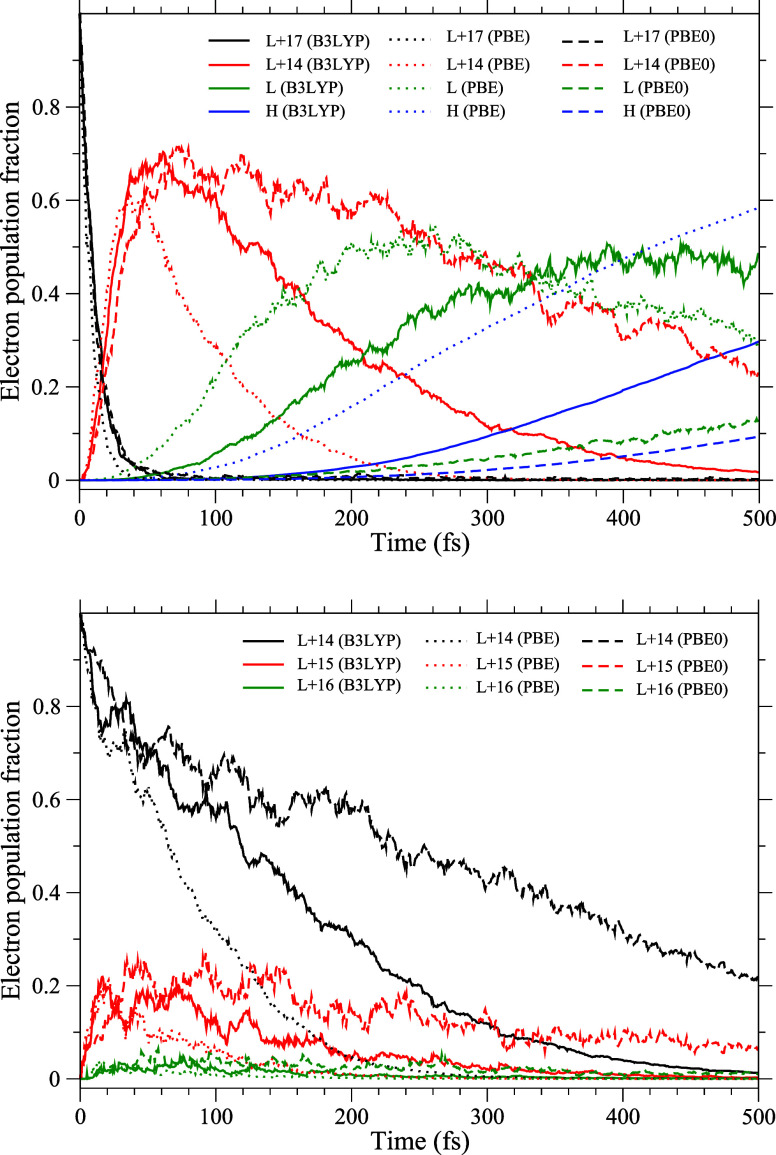
Top: Time evolution of the population of initially excited LUMO+17 and intermediate LUMO+14 state and the partial evolution of LUMO and HOMO (recombination) state calculated using the B3LYP, PBE and PBE0 functionals. Bottom: Same as top, but for the initial LUMO+14 state and subsequently sputtered population to higher LUMO+15 and LUMO+16 states.

One general goal in this section is to directly simulate the transient electron capture dynamics at the top edge of an energy gap and use this dynamic advantage to probe the electron promotion to higher states. We focus on the gap below LUMO+14 (Figure 1). First, an initial excitation to LUMO+17 is considered so that we can scrutinize the promotion to LUMO+15 and 16. The top panel of Figure 3 displays the simulated population time-evolution of the initially excited (100% population at time zero) LUMO+17 state in three xc functionals. As seen, LUMO+17 depopulates in roughly 50 fs in all three treatments, while PBE exhibits the fastest decay. As the electron decays to LUMO+14, it experiences a transient entrapment due to a wide energy gap below this state, which slows its subsequent decay. This effect is found to be universal in the three xc approaches. The population dynamics of any intermediate state involve a combination of growth and decay dynamics. As a result, for LUMO+14, the growth due to the electron transfer from higher states dominates during earlier times to peak the cumulative population to 60% or above. Subsequently, the decay begins to dominate and continues over remarkably longer times. It should be noted that PBE0 yields the highest peak population, which also experiences the longest decay period. B3LYP closely follows up to the peaking time, although later decaying relatively faster. On the other hand, PBE features a slightly smaller maximum population with a significantly fast decay trail. These details are partly a direct ramification of differences in the size of the gap immediately below LUMO+14 in three xc approaches (Figure 1). However, there are more aspects regarding this, which will be discussed in Subsections 3.3 and 3.4. The similar comparative trend in the dynamics of LUMO at the band-edge, as well as the return of the population to HOMO (recombination), among the results of three xc functionals, is also seen in this figure for completeness. It may be noted that the peak population growth is not captured for LUMO in PBE0 and for HOMO in all three methods within the displayed range of up to 500 fs.

To further examine the procrastinated decay of the LUMO+14 state, we present its dynamics in the bottom panel of Figure 3 by choosing this state to be the initially excited state. The purpose here, as pointed out above, is to probe the electron promotion to two higher states and subsequent decay. As seen, the general comparative temporal behavior among the methods discussed above is clearly retained. That is, PBE0 induces the slowest decay, while PBE induces the fastest. This figure further discloses another feature of the mechanism that contributes to the dynamics. This entails some population of LUMO+14 to be *promoted* to LUMO+15 and even to LUMO+16. This highlights the role of the nuclear-vibration-driven electron dynamics within the quasi-degenerate, compact states. As seen, due to the longer holdup of electrons in LUMO+14, some of the probability transitions are higher in energy to LUMO+15 to populate it up to a maximum of about 20% both in PBE0 and B3LYP, while to a lesser extent in PBE. A similar behavior is seen for LUMO+16 as well but on a much smaller scale. This “sputtered” electron density will return to *repopulate* LUMO+14–an effect that further favors LUMO+14’s delayed decay. An identical behavior of the C_60_ LUMO+14 level was earlier found in our study of Mg to C_60_ ultrafast CT relaxation in the endohedral Mg@C_60_.^29^ It should be noted further that the decay of the population promoted to LUMO+15 is slow as well, due primarily to the congestion caused by the energy gap, although it readily repopulates LUMO+14.

### 3.3 Nonadiabatic Couplings

The electronic wave function of the molecule can be expressed by a time-dependent linear superposition of molecular orbitals ϕ_*j*_ with, say, *C*_*j*_ being the mixing coefficients that act as the electronic degrees of freedom. *C*_*j*_ must evolve by the time-dependent Scrödinger equation in natural units as
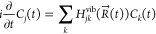
1Here the vibrionic Hamiltonian matrix *H*^vib^ can be written as
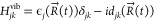
2where  is the nuclear coordinate, and δ_*jk*_ are Kronecker delta symbols. The computed ϕ_*j*_ and orbital energies ϵ_*j*_ along the nuclei trajectories are used to obtain NACs *d*_*jk*_:^52^
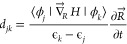
3where *H* is the electronic Hamiltonian. Evidently, NACs can be enhanced by (i) larger orbital overlaps, (ii) narrower energy separations, and (iii) faster nuclear velocities.

**Figure 4:**
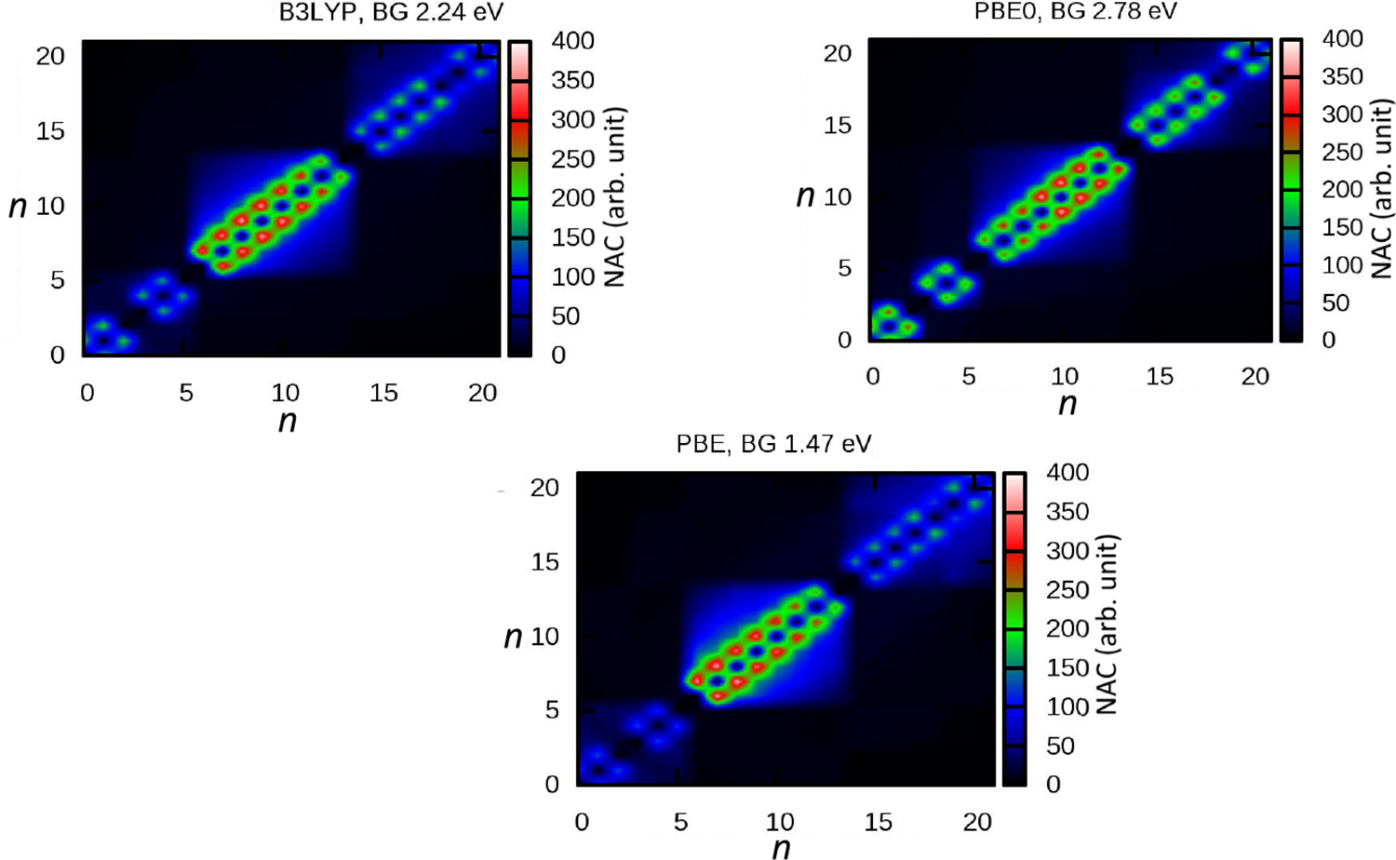
Magnitudes of MD-averaged nonadiabatic couplings (NACs) calculated using the B3LYP, PBE0 and PBE functionals and plotted in the arbitrary unit universal among three panels. The axes plot the index *n* of the LUMO+*n* states. The band gap (BG) values are found after the NVE simulation and thus slightly differ from the values quoted in Figure 1.

Figure 4 presents the trajectory-averaged magnitudes of NACs involving couplings among LUMO to LUMO+22 states in the three xc frameworks employed. The diagonal trace shows zero signals due to the nonexistence of self-coupling. The super- and subdiagonal traces represent predominant signals owing to the strongest couplings between nearest neighboring levels. It should be noted that universally in all three cases, the NAC signals between states LUMO+14 and LUMO+13, LUMO+6 and LUMO+5, and LUMO+3 and LUMO+2 pairs are very weak, appearing practically dark in the color scale of Figure 4. Obviously, this is due to the large energy difference, via the denominator of eq 3, from the gap between these states, indicating their very weak mutual NAMD transition. Besides, as seen, there are differences in the finer details of NAC values among xc methods. These differences must be due to the differences in the details of interorbital overlaps based on variations in the structure of molecular orbitals and nuclear velocities among the xc functionals. All these effects will collectively determine variations in NACs as a function of the xc scheme and will have consequences in the dynamics they drive. We showcase some representative results and comparisons between B3LYP and PBE0 in the following subsection.

### 3.4 Decay and Transient-Capture Times

We use the following fitting scheme to determine average evolution times.^44,53^ The temporal decay of the population fraction of the initially excited state is fitted to the sum of an exponential and a Gaussian decay function appropriately weighted as
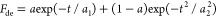
4where *a* is the weight parameter with a value between 0 and 1, and *a*_1_ and *a*_2_ are antisteepness parameters such that the average decay time (τ_de_) is evaluated by
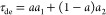
5The evolution of the population fraction for an intermediate state, on the other hand, will involve a combination of both growth and decay processes. To stay consistent with eq 4, yet to introduce a different set of parameters, a fit formula for the growth component can be written as
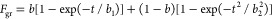
6with 0 < *b* < 1. Thus, a general fitting formula for an intermediate state, affected by both decay and growth, can be considered as
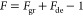
7with the average decay time
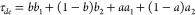
8

**Figure 5:**
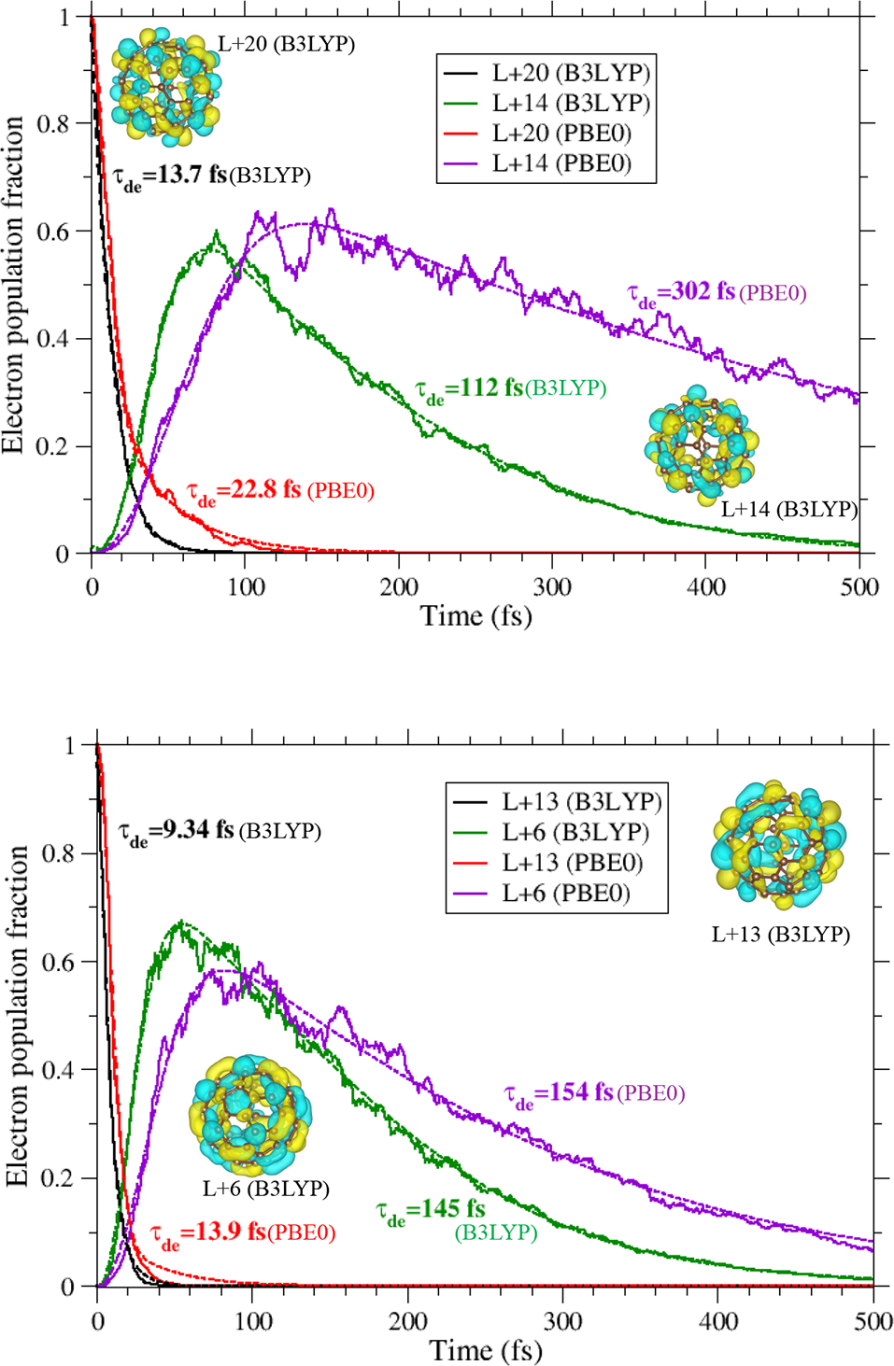
Top: Time evolutions of the populations of initially excited LUMO+20 and intermediate trapper state LUMO+14 were calculated by B3LYP and PBE0. The noted average decay times (τ_de_) are extracted by curve fittings (see text) and the fit curves for the decay are plotted by the dashed lines. The DFT/B3LYP generated isosurface orbital plots are illustrated. Bottom: Same as top, but for the initial LUMO+13 states and the trapper state LUMO+6.

The NAMD simulation of the initially excited LUMO+20 state and the transient trapper state LUMO+14 is compared for B3LYP and PBE0 in Figure 5 (top panel). LUMO+20 safely ensured the highest initial excitation within the set of LUMO levels, up to LUMO+22 (Figure 1) employed in the active space, so that the two remaining higher levels can sufficiently account for the return of the promoted population. Fitting with eq 4 produces 13.7 and 22.8 fs of average decay times (eq 5) for LUMO+20 in, respectively, B3LYP and PBE0. An important mechanism that can influence a slower decay is the process of repopulation of the state. This includes the return of electron population from the lower energy states where the electron decayed. The higher probability of repetition of this cycle will effectively sustain the net electron population longer. Therefore, the NACs govern these transition rates – the higher the value of the NAC the stronger the rate. As seen in Figure 4, PBE0 NACs involving states from LUMO+20 and closely below are slightly stronger than those of B3LYP suggesting a more sustained back-and-forth of electrons producing a longer decay time in PBE0. Furthermore, this difference is more pronounced for the decay of LUMO+14 that features, upon fitting with eq 6, an average time (eq 8) of 112 fs in B3LYP and 302 fs in PBE0! Of course, a slightly larger gap below LUMO+14 in PBE0 (Figure 1) favors this effect. But the more complete reason for this difference is again the larger NAC values in PBE0 involving LUMO+14 and states above it that ensure significant excitation to higher states in PBE0; this was qualitatively addressed earlier in the context of Figure 3. This sputtered population to higher states keeps feeding LUMO+14 back in order to facilitate the rise of its population. This population maximizes until roughly 50 fs later in PBE0 than B3LYP, as seen. Therefore, stronger NACs in PBE0 result in LUMO+14’s rather significantly longer decay time with this functional. It is therefore obvious that even though the LUMO energy distribution is identical in B3LYP versus PBE0, the differences in interorbital overlaps and nuclear velocities can affect significantly varied dynamics via altered NACs.

Figure 5, bottom panel, explores a similar NAC-induced mechanism but for some lower states. This figure considers LUMO+13 as the initial excited state and also monitors the population of intermediate LUMO+6, which has another energy gap below it (Figure 1). The choice of LUMO+13 was to consider another test case but with two lower LUMO gaps (Figure 1), so as to compare with the results above. The gap immediately above LUMO+13 in effect forbids promoted population. Maintaining the trend, LUMO+13 decays slower with an average of 13.9 fs in PBE0 than 9.34 fs in B3LYP. Evidently, this difference is owed to the larger NAC values in PBE0, versus B3LYP, for states immediately below from LUMO+13 (see Figure 4). Remarkably, this trend reverses for NACs involving LUMO+6 and above, on the other hand, with PBE0 showing weaker values than B3LYP – an effect that reduces the degree of repopulation of LUMO+6 in PBE0 than B3LYP. Indeed, the B3LYP peak in this case rises higher up to barely below 70%, even though it still occurred 20 fs earlier. Consequently, the LUMO+6 average decay times produced by two functionals come very close being 145 fs (B3LYP) and 154 fs (PBE0). We note that the energy gap below LUMO+6 is slightly larger in PBE0 which somewhat hinders its decay across the gap more.

A broad picture of the NAMD decay mechanism, irrespective of the chosen xc scheme, can now be drawn, which is an aggregated account of three processes: (i) the direct population decay to energetically lower states (a loss), (ii) the population back-transfer from those lower states (a gain), and (iii) the return from the population promoted to energetically higher states (another gain). For a state just above an energy gap, like LUMO+14 and LUMO+6, (i) and (ii) above are very weak compared to (iii) leading to their significantly longer decay times. On the other hand, for a state just below a gap, say LUMO+13, the situation is quite the opposite with (iii) being too weak and (i) too strong resulting in their shortest decay times. Interestingly, for a state in the middle of an energy band, like LUMO+20, (iii) is not so weak in dealing with states above it within the band, while (i) is still strong. Consequently, the LUMO+20 decay time will be slightly longer than LUMO+13, exactly as found and displayed in Figure 5. Hence, state-selected ultrafast spectroscopy can access information that can map out some details of the LUMO structure. In dynamics based on the MB scheme, the initially excited state should be selected as a bright state where the oscillator strength is large. While our method relies on an IP scheme. However, the effects uncovered and described above are rather fundamental and robust and thus are expected to survive even for somewhat energetically shifted but otherwise bright transitions.

We have noted, and as the trend of Figure 3 also suggests, that the PBE-derived dynamics (results not included) is generally the fastest among the three methods. This can be understood from the following observations in the PBE NACs in Figure 4. (i) The higher level PBE NACs involving LUMO+20 and the states below are (slightly) weaker than those of B3LYP. (ii) The NAC values for LUMO+13 and below in PBE exhibit a trend similar to that of B3LYP when compared to PBE0, although the high-end part in PBE is even weaker than B3LYP. (Incidentally, this general trend of NACs calculated in a nonhybrid functional like PBE being weaker than those obtained in hybrid functionals was suggested earlier^54^). Moreover, as found, all of the energy gaps in PBE are narrower than in other methods (Figure 1). All these have a net influence in the reduction of repopulation resulting in a faster overall decay in PBE. Even though the results of hybrid B3LYP and PBE0 are likely more accurate, the inexpensive PBE can yet capture qualitatively the correct trends.

## 4 Spectrogram for Experiment

**Figure 6:**
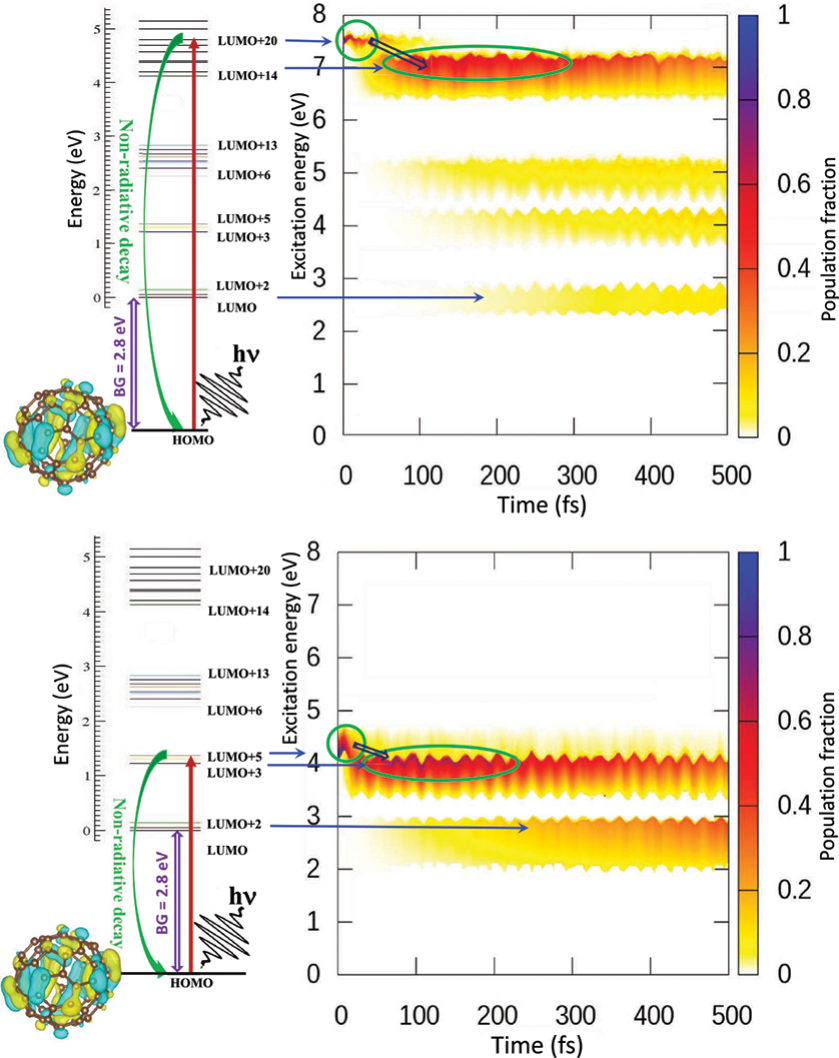
Top: The decay dynamics of initially excited LUMO+20 through all transient states produce a time-energy spectrogram contour map of the population fraction calculated in PBE0. The excited population decay of LUMO+20 to a transient capture in LUMO+14 (indicated) and final population buildups in lower states up to LUMO are shown in a color coded spectrogram. The energy gaps are clearly mapped by showing zero populations. Note that while in the LUMO energy band on the left the LUMO energy is set to zero, HOMO is considered zero on the spectrogram, rendering this the excitation energy scale. The HOMO–LUMO band gap (BG) and the HOMO isosurface orbital image are shown. Bottom: Same as top but for the initial excitation to LUMO+5 and transient capture in LUMO+3.

The ultrafast relaxation of pump laser-pulse-excited C_60_ molecules, in vapor or condensed matter phases, can be followed by a time-delayed probe pulse in the TRPES experimental track. The measurements can produce two-dimensional spectral information (spectrogram) as a function of the pump energy and pump–probe time delay.^7,17−19^ While the current study does not include the probe effect, the NAMD-based *ab initio* relaxation approach incorporated ensures that the result is generally robust. The effects are therefore expected to dominate the TRPES signal since the probe pulse is conventionally weak. Thus, at the very least, a strong qualitative similarity of the NAMD time-dependent population maps can be expected with an experimental spectrogram. Figure 6 includes such contour maps of the excited and transient electronic state population dynamics obtained from the DFT trajectories in our simulations for the LUMO+20 and LUMO+5 initial pumped states. To illustrate, we only use results obtained by the PBE0 framework. It should be noted that a visual similarity of the HOMO orbital image in PBE0 in this figure to that in Figure 1 is calculated in B3LYP.

Figure 6, top panel, delineates transient electronic populations following the excitation from HOMO to LUMO+20 state, as in Figure 5, by a far UV pump. In this map, the color-coded (*z*) direction accounts for the dynamic population fraction. Therefore, the population curve for each excited LUMO+*n* state is on the *xz* plane corresponding to the LUMO energy in *y*. Note that from LUMO+20, the hot electron quickly decays through the states below to transiently confine in LUMO+14 above the energy gap for an extended time. A similar trend is qualitatively repeated for the states LUMO+6, LUMO+3, and LUMO, each top-edging a gap. However, a somewhat reduced population is recorded going progressively further from LUMO+20 within the 500 fs time window shown. This slowdown toward the band edge is due to additional slowing effects induced during the decay across multiple gaps. We remark that the oscillations in time noted in the contour plot are due to lattice motions on the ground state potential energy surface coupled to the electron motion. It may be noted that the current study is based on the so-called neglect-of-back-reaction approximation,^55^ where the nuclear evolution is not affected by changes in the electronic states, implying trajectory runs on the ground state molecular dynamics.

Figure 6, bottom panel, exhibits similar dynamics but for a mid-UV excitation to a lower LUMO+5 level. This initial excitation was chosen to examine the dynamics directly across the lowest LUMO gap (Figure 1). On both panels, the very light-shaded regions correspond to extremely weak and fast decay through the compact energy band levels below the bottom edge of the gaps. Also, some signal encroaching into the gaps is a numerical artifact due to the interpolation from strong population transiency at the gap top.

To summarize, within the reliability of a robust and ab initio methodology in DFT, the prediction of strong population traps atop the energy gaps on the decay path appears to be plausible and has a fundamental effect. The fact that all these dynamics are reasonably captured as population growth and decay traces in Figure 6 bodes well for TRPES-type measurements in accessing the structure of the fullerene excited states in general and probing dominant effects in particular. Experiments can further assess the relative accuracy of B3LYP versus that of PBE0.

## 5 Conclusions

The ultrafast nonradiative relaxation process, driven by electron–phonon coupling (lattice thermalization), of a photoexcited electron in molecular C_60_ is simulated in two hybrid xc functionals, B3LYP and PBE0, within the DFT framework. Insuring reliance, the LUMO energy structure is found to compare reasonably well with the many-body excited state structure. The ultrafast relaxation results are compared and analyzed, which sheds light on the simultaneity of the decay, promotion, and repopulation processes from vibrionic motions in determining the dynamics. The study features a transient slowdown phenomenon, on the order of one hundred to a few hundreds of femtoseconds, of the relaxation process in real-time due to the presence of gaps in the spectrum of excited electronic states. The systematic trend of the decay at the gap bottom being fast, on the order of tens of femtoseconds, with progressive slowdown reaching up to the band top demonstrates a temporal route to tap in the excited state structural information by ultrafast spectroscopy. Ideally, the separation of quantum mechanical nuclear wave functions will reduce the coherence between electronic states^56,57^ – a feature that is missing in our current semiclassical model of nuclear dynamics and is the topic of an upcoming study.^58^ It will further be interesting to know how the dynamics, in general, and the sizes of the LUMO gaps, in particular, quantitatively evolve in an electron–hole coupled-configurations framework.

To this end, however, the effects found appear robust and fundamental, particularly given that results deploying a far inexpensive nonhybrid PBE functional yielded similar trends. The quantitative temporal information alters from one xc model to another with PBE being the fastest and PBE0 the slowest but closer to B3LYP. This is based on the integrated effects of unoccupied band energy structure, interorbital overlaps, and nuclear vibrational velocities, all of which evolve as a function of the model.

The study provides motivation to conduct two-photon pump–probe UTAS or TRPES measurements on fullerene molecules, which are stable and symmetric and can be prepared in the vapor phase for experiments relatively easily. The measured spectrograms can be directly compared to the contour maps that can be simulated in our methods. The comparison will probe the current predictions. It will also help identify the best-performing xc scheme in order to optimize the computational methodology. Such optimization will be of great value to extend the study for the investigation of fullerene derivatives with increasing structural complications via endohedral/exohedral doping, functionalization, and polymerization. We hope that the current results will pave the way for experimental efforts in the domain of ultrafast science to complement our ongoing theoretical campaign.
